# Domenico Mistichelli (1675–1717)

**DOI:** 10.1007/s00415-024-12727-0

**Published:** 2025-01-07

**Authors:** Jan van Gijn, Stefano Sandrone

**Affiliations:** 1 Department of Neurology, University Medical Centre Utrecht, Utrecht, The Netherlands; 2 Department of Brain Sciences, Imperial College London, London, UK

**Keywords:** History of neurology, History of neuroscience, Domenico Mistichelli, Pyramidal crossing

Domenico Mistichelli, about whose life rather little is known, identified the site of what is nowadays known as the ‘pyramidal crossing’. That paralysis or convulsive movement on one side of the body is associated with a brain lesion on the contralateral side is found in the Hippocratic writings [1]. Later, Greek physicians perpetuated it. One of them, Aretaeus of Cappadocia (late 2^nd^ century CE), also knew that the crossing did not occur with lesions of the spinal cord, so he concluded that some interchange of nerves should take place:

*Then, if some point of origin is affected below the head, such as the membrane of the spinal cord, the homonymous and connected body parts will be paralysed – the right ones on the right and the left ones on the left. But if the provenance is in the head, the body parts on the left are paralysed with lesions on the right side, and body parts on the right with those on the left side. The cause is a crossing at the beginnings of the nerves; [...] close to their origin either set moves straight to the other side, crossing each other in the form of the letter X* [2]*.* (NB translation from Greek by JvG)

This part of the Greek medical tradition was almost forgotten in Western Europe until it was rekindled in the ‘medical renaissance’ of the sixteenth century [3]. Therefore, the ‘Hippocratic crossing’ was widely accepted in several universities, certainly in Italy, though Giovanni Battista Morgagni (1682–1771) attributed the ‘law’ to his teacher Antonio Maria Valsalva (1666–1723) [4]. Nevertheless, some physicians made strange mistakes in identifying the side of the brain lesion in patients with paralysis [5].

Mistichelli was born on 10 January 1675 in Fermo, in the south of the Marche region [6]; his family came from nearby Monte San Pietrangeli. He studied medicine, like his grandfather, at the Universities of Fermo and Macerata [6]. Two streets in the region are now dedicated to him. After graduation, he moved to Rome, where he practised as a physician and teacher at the *Ospedale della Consolazione*, and was appointed personal physician of Cardinal Niccolò Acciaiuoli, Dean of the Sacred College [6].

**Fig. 1:**
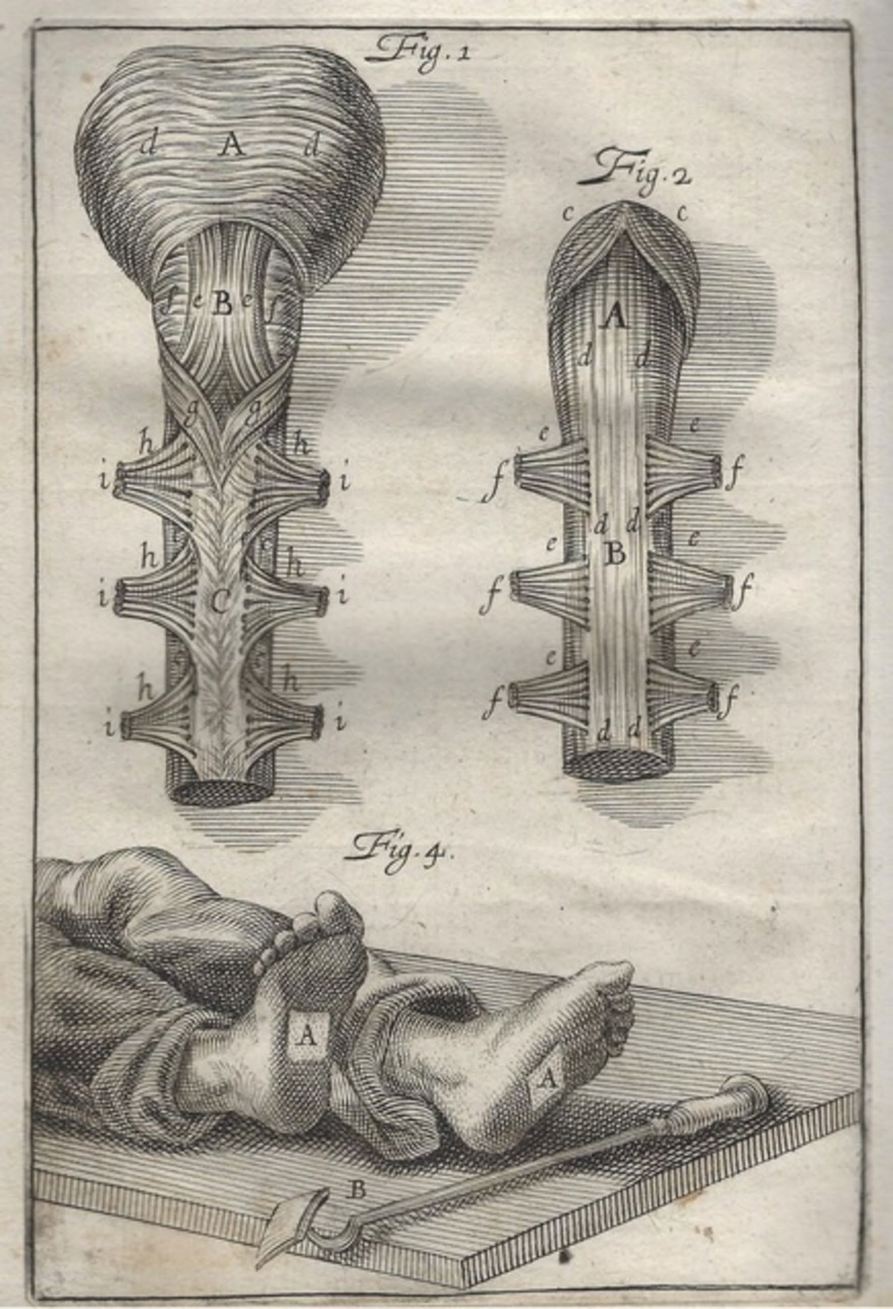
Original drawings from Mistichelli [7]: ‘Fig. 1’: Ventral aspect of medulla oblongata and spinal cord. ‘Fig. 2’: Dorsal aspect of medulla oblongata and spinal cord. ‘Fig. 4’: The branding iron and the site (A) where the branding iron should be applied to the foot

Mistichelli’s anatomical observations form part of his book on apoplexy, published in 1709 [7]. In the Preface, he explained that he had started writing in 1705 and 1706, when employed in Rome at the *Ospedale Fatebenefratelli* on the Tiber Island, as an adjunct to the influential professor Giovanni Maria Lancisi (1654–1720). Lancisi had been commissioned by Pope Clement XI to investigate the many sudden deaths occurring in Rome. Mistichelli initially suppressed his own text, to give precedence to Lancisi’s book [8]. However, later advice and new observations induced him to publish after all, citing Pliny the Younger’s adage “No book is so bad that it is not profitable in some detail.” Yet, it seems his relationship with Lancisi became troubled afterwards [6]. Mistichelli wrote in Italian (Tuscan) rather than in Latin, because he wished to inform both the public and physicians. Though he had read works on apoplexy by sixteenth-century authors, for him, the terms ‘apoplexy’ and ‘sudden death’ largely overlapped. Consequently, he implicated changes in organs other than the head as potential causes of apoplexy. He was more a surgeon and anatomist than a pathologist [9]. The few autopsies of patients with brain lesions he performed or attended did not lead to any new insights. A new and remarkable advice on treating apoplexy was to apply a branding iron to the sole of the patient’s foot (fig. 1).

As an anatomist, Mistichelli had been struck by a configuration of nerve fibres on the dorsal side of the upper medulla (fig. 1). He first described the actual crossing of the nervous fibres, ‘similar to the plait of a Lady’.

*The most notable feature is the interlacement* [intrecciamento] *of the fibres of the membranes that hem it in. Part of the medulla oblongata and of the spinal medulla coated by its membranes is kept in an acetic solution for eight and ten days and after these have expanded to the size of a mid-sized spine of a knife, first a delicate removal takes place of the blood vessels wound around it (running along the length of the spinal cord in the form of a net-like covering), and subsequently of the outer layer of fibres, which form the said membranes. Once the inside and the innermost layer have been reached, one observes that the entire brain stem* [caudice]* on the outside might resemble the plait of a Lady because numerous groups of straight fibres are superimposed on many transverse fibres, many oblique ones on transverse ones and other straight ones; by following this interlacement, every part, in turn, covers or is covered, until the said fibres go out from the Plait to form the spinal nerves, which are on the sides.*

He perceived this configuration could explain the curious phenomenon described by Hippocrates:

*To explain these [Hippocratic] texts, it is necessary to recall what we have observed as a novelty: the medulla oblongata is on its outside covered by fibres that are mutually interwoven and represent a lady’s braid; hence it occurs that many nerves branching out to one side contain the roots of another side. For example, the [nerves] extending to the right arm can easily contain, by this interlacement, roots from the fibres on the left side of the meninges; the same applies to the left ones, proceeding from the right side* [7]*.*

The reader may have noticed that Mistichelli, like the ancient Greeks, regarded the meninges as an integral part of the nervous system. One year after his book appeared, the Parisian Pourfour du Petit (1664–1741) published a more accurate description and illustration of what he then called the ‘pyramidal bodies’ [10]. Yet, it is Mistichelli who should be remembered for first drawing attention to the site where descending motor fibres cross. In the last years of his life, he moved to Ancona, where he died on 22 September 1717, at 42 years of age [6].
